# Financial Analysis of Herd Status and Vaccination Practices for Porcine Reproductive and Respiratory Syndrome Virus, Swine Influenza Virus, and *Mycoplasma hyopneumoniae* in Farrow-to-Finish Pig Farms Using a Bio-Economic Simulation Model

**DOI:** 10.3389/fvets.2020.556674

**Published:** 2020-11-09

**Authors:** Julia Adriana Calderón Díaz, Rose Mary Fitzgerald, Laurence Shalloo, Maria Rodrigues da Costa, Jarkko Niemi, Finola C. Leonard, Ilias Kyriazakis, Edgar García Manzanilla

**Affiliations:** ^1^Pig Development Department, Teagasc Animal and Grassland Research and Innovation Centre, Fermoy, Ireland; ^2^Bio-Explore, Department of Biological Sciences, Cork Institute of Technology, Bishopstown, Ireland; ^3^Livestock Production Systems, Teagasc Animal and Grassland Research and Innovation Centre, Fermoy, Ireland; ^4^School of Veterinary Medicine, University College Dublin, Dublin, Ireland; ^5^Natural Resources Institute Finland (LUKE), Seinäjoki, Finland; ^6^Institute for Global Food Security, Queen's University of Belfast, Belfast, United Kingdom

**Keywords:** herd health status, pig production systems, respiratory diseases, whole-farm stochastic budgeting, financial risk

## Abstract

This study aimed (1) to quantify the effects of positive status and vaccination practices for porcine reproductive and respiratory syndrome virus (PRRSv), swine influenza virus (SIV) and *Mycoplasma hyopneumoniae* (MHYO) on the profitability of farrow-to-finish pig farms and (2) to examine the financial impact of vaccination status in PRRSv and SIV positive farms. Data from 56 Irish farrow-to-finish pig farms were used for this study. Production effects associated with herd status for the three pathogens were incorporated into the Teagasc Pig Production Model (TPPM), a bio-economic stochastic simulation model for farrow-to-finish pig farms. In the analysis, farms negative (–) for either PRRSv, SIV or MHYO were assumed as baseline when presenting results for farms positive (+) for each pathogen. While all MHYO(+) farms used vaccination against the pathogen, not all PRRSv(+) or SIV(+) farms vaccinated against the disease. For all scenarios, a 728-sow farrow-to-finish farm with weekly farrowing batches was simulated. Financial risk analysis was conducted by Monte Carlo simulation within the TPPM using the Microsoft Excel add-in @Risk. Mortality rates, feedstuff costs and price per kg of meat produced were included as input stochastic variables and annual net profit was set as stochastic output variable. Positive farms sold fewer pigs and produced less kg of meat than negative farms and had increased feed usage during the weaner and finisher stages. Variable costs increased in positive farms due to increased feed costs, more dead animals for disposal and healthcare costs. Annual mean profit was lower by 24% in vaccinated PRRSv(+), 14.6% in unvaccinated PRRSv(+), 36.7% in vaccinating SIV(+), 12.8% in unvaccinated SIV(+), and 41% in MHYO(+) farms. Negative farms were first order stochastically dominant over positive farms, indicating that for a given level of profit, the financial risk is lower by avoiding respiratory pathogens. Similarly, unvaccinated farms were second order stochastically dominant over vaccinating farms suggesting that farms that do not vaccinate are less affected by the disease. Results from this study provide further evidence to encourage farmers to undertake improved disease control measures and/or to implement eradication programs.

## Introduction

Porcine reproductive and respiratory syndrome virus (PRRSv), swine influenza virus (SIV) and *Mycoplasma hyopneumoniae* (MHYO) are among the most common agents involved in the porcine respiratory disease complex (1). They are also among the most significant infectious conditions contributing to substantial losses in the pig industry (2–4). Financial losses are mainly attributed to increased mortality rates, increased feed costs, reduced growth performance and increased costs for vaccination and disease control (5–9).

Porcine reproductive and respiratory syndrome virus is present in most pig producing regions; it was first reported in the Republic of Ireland in 1999 (10). It is transmitted by several routes including intranasal, oral, intrauterine and vaginal (11), and it tends to become endemic once introduced to the herd (12). In infected herds, regular or occasional outbreaks are observed in weaner and finisher pigs (13) decreasing average daily gain and increasing feed conversation ratio (14). Porcine reproductive and respiratory syndrome virus is also associated with reproductive failure, mainly manifested as return to oestrus and abortions, non-pregnant gilts and abnormal litters (11, 15).

Swine influenza virus outbreaks are associated with high fever and loss of appetite, abortions, and other reproductive problems (5, 16). Although mortality is low and most animals recover (17), SIV has a financial impact mainly due to decreased average daily gain in infected pigs (5, 6). In temperate climates, SIV outbreaks occur mostly during the fall and winter seasons when temperatures start to drop (16); although SIV circulates year round (18). It is commonly introduced to pig farms through animal movement and the main route of transmission is through nasopharyngeal exposure (16). Vaccination of breeding females is a common strategy used to protect offspring from SIV by improving maternal antibodies and providing them with early immunity (19).

*Mycoplasma hyopneumoniae* is the main agent of enzootic pneumonia in pigs and it is transmitted by nose-to-nose contact between infected and susceptible animals (20). Infection persists for prolonged periods (21) and affected pigs can remain infectious for up to 200 days (22). Infected pigs have decreased growth performance and can take, on average, 5 days longer to reach slaughter weight compared to unaffected animals (9). Vaccination is commonly used to reduce clinical signs, decrease infection levels and improve performance (23); however, vaccination confers only limited protection against transmission of MHYO (24).

Although many authors state PRRSv, SIV and MHYO cause important financial losses, there are few reports in the scientific literature quantifying such losses and most of these are based on data from USA pig farms. For instance, Holtkamp et al. (8) estimated that PRRSv is responsible for economic losses of US$668.6 million annually in the USA pig industry and Dee et al. (25) estimated that endemic PRRSv costs at least US$10.5 per pig produced in farrow-to-wean production systems. In the case of a PRRSv outbreak, Nieuwenhuis et al. (12) estimated mean financial losses of €126 per sow in Dutch sow herds. Haden et al. (6) estimated a financial loss of US$3.23 per pig produced associated with SIV infection while Donovan (5) estimated a higher value of US$10.31 per pig produced in a wean-to-finish production system in the USA. For MHYO, Haden et al. (6) reported a financial loss of US$0.63 per pig produced in a wean-to-finish production system in the USA. Specific assumptions incorporated into North-American studies mean that the results are not easily comparable to Europe. Additionally, all previous studies used a deterministic modeling approach whereby the unpredictable impact of respiratory disease was not taken into consideration. This study aimed to conduct a bio-economic analysis, for the first time using actual data from commercial farms, on the effects of herd status and vaccination practices for PRRSv, SIV and MHYO on the profitability of farrow-to-finish pig farms using a previously constructed stochastic bio-economic model.

## Materials and Methods

### Disease and Vaccination Status

Data on disease status and vaccination practices for PRRSv, SIV, MHYO, and *Actinobacillus pleuropneumoniae* (APP), were previously obtained by Rodrigues da Costa et al. (*submitted*) in a cross-sectional study in 56 Irish farrow-to-finish pig farms. In short, all farms providing data to the Teagasc e-Profit Monitor system in 2017 (*n* = 107) were initially contacted directly by telephone or by their Teagasc pig advisor and invited to participate in the study. A total of 56 farmers voluntarily agreed to participate (52.3% compliance). Upon receiving written consent from the farmers, blood samples were collected from November 2017 to April 2018 from a total of 32 randomly selected finisher pigs per farm at exsanguination at slaughter. Samples were transported for analysis to the Blood Testing Laboratory of the Department of Agriculture Food and the Marine (Cork, Ireland). Serum samples were analyzed using enzyme-linked immunosorbent assay (ELISA) according to the manufacturer's instructions (IDEXX, Hoofddorp, The Netherlands) for the four respiratory pathogens. Sample-to-positive ratio values were calculated for APP, PRRSv and MHYO while sample-to-negative ratio values were calculated for SIV. Samples with sample-to-positive values ≥ 0.40 for PRRSv and MHYO, ≥ 0.50 for APP and samples with sample-to-negative values SIV ≤ 0.60 were considered as positive as per the criteria given in the manufacturer's instructions. Then, farms were considered positive to any of the four respiratory pathogens if at least one animal tested positive in the ELISA test. To our knowledge, no new disease outbreaks were reported in any of the farms during the year 2017 and until April 2018 for any of the studied diseases. Vaccination practices for each farm for the year 2017 were obtained through phone calls to farmers and their corresponding private veterinary practitioners during the same time period. The information collected included disease vaccinated for and production stage where vaccine was used.

### Scenarios

Farm net profit was studied in five scenarios depending on herd status and vaccination strategy for PRRSv, SIV, and MHYO. Sample size was estimated at 10 farms per scenario based on a difference in average daily gain of 40 g between negative and positive farms. Average daily gain was selected to calculate sample size based in previous results from our research group and on previous reports from the scientific literature regarding decreased growth performance associated with the three studied pathogens (5, 6). A *t*-test for two-group independent sample was used to calculate sample size in PROC POWER of SAS v9.4 (SAS Inst. Inc., Cary, NC). The scenarios are described below:

- Unvaccinated PRRSv negative [PRRSv(–)] farms (*n* = 23) compared with PRRSv positive [vacPRRSv(+)] farms (*n* = 19) vaccinating gestating sows at ~60–80 days of gestation with a single dose. Maiden gilts were vaccinated before entering the breeding herd. Farms vaccinating only weaner pigs (*n* = 5) were not used for building a new scenario due to the low number of observations.
- Unvaccinated PRRSv negative [PRRSv(–)] farms (*n* = 23) compared with unvaccinated PRRSv positive [unvacPRRSv(+)] farms (*n* = 9).
- Unvaccinated SIV negative [SIV(–)] farms (*n* = 11) compared with SIV positive farms (*n* = 20) vaccinating [vacSIV(+)] gestating sows at ~60–80 days of gestation with a single dose. Maiden gilts were vaccinated before entering the breeding herd. One SIV negative farm and one SIV positive farm vaccinating sows and piglets were not used for analysis.
- Unvaccinated SIV negative [SIV(–)] farms (*n* = 11) compared with unvaccinated SIV positive [unvacSIV(+)] farms (*n* = 23).
- Unvaccinated MHYO [MHYO(–)] negative farms (*n* = 10) compared with MHYO [MHYO(+)] positive farms (*n* = 39) vaccinating pigs at weaning (i.e., 28 days of age) with a single dose. Negative farms vaccinating (*n* =2), positive farms vaccinating sows and piglets (*n* =1), positive farms vaccinating sows (*n* =1) and positive farms not vaccinating (*n* = 3) were not used for analysis.

For all the scenarios, all pigs were vaccinated for porcine circovirus type 2 at weaning. Also, maiden gilts and lactating sows were vaccinated for *Erysipelothrix rhusiopathiae* and porcine parvovirus as per normal practice in Irish pig farms.

The number of farms positive and negative used for the different scenarios for each of the three infections and their combinations and their vaccination status is given in Table 1. It was not possible to simulate co-infection scenarios as there were few farms negative for two (i.e., 9 farms) or all three (i.e., 4 farms) pathogens. Thus, farms PRRSv(-) could be positive for MHYO and/or SIV and farms PRRSv(+) could also be positive to MHYO and/or SIV. A similar situation was possible for the other scenarios. Additionally, 55 of the 56 farms were positive for APP.

**Table 1:** Number and percentage (%) of positive and negative farms and farms vaccinating for each of three respiratory pathogens [porcine reproductive and respiratory syndrome virus (PRRSv), swine influenza virus (SIV) and *Mycoplasma hyopneumoniae* (MHYO)], and their combinations within eight different farm classifications to investigate the effect of herd status and vaccination practices for PRRSv, SIV, and MHYO on the profitability of farrow-to-finish pig farms.

	**PRRSv**		**SIV**		**MHYO**		**PRRSv** **+** **SIV**		**PRRSv** **+** **MHYO**		**SIV** **+** **MHYO**	
**Farm classification**	***n***	**%^**a**^**	***n***	**%**	***n***	**%**	***n***	**%**	***n***	**%**	***n***	**%**
**FARMS POSITIVE**												
PRRSv negative^b^ (*n* = 23)	–	–	14	60.9	16	69.6	–	–	–	–	11	47.8
PRRSv positive^c^ vaccinated (*n* = 19)	19	100	19	100	18	94.7	–	–	–	–	18	94.7
PRRSv positive^b^ unvaccinated (*n* = 9)	9	100	6	66.7	5	55.6	–	–	–	–	4	44.4
SIV negative^b^ (*n* = 11)	2	18.2	–	–	6	54.5	–	–	1	9.1	–	–
SIV positive vaccinated^c^ (*n* = 20)	17	85.0	20	100	18	90.0	–	–	16	80.0	–	–
SIV positive unvaccinated^b^ (*n* = 23)	13	56.5	23	100	19	82.6	–	–	11	47.8	–	–
MHYO negative^b^ (*n* = 10)	4	40.0	4	40.0	–	–	2	20	–	–	–	–
MHYO positive^d^ vaccinated (*n* = 39)	27	69.2	35	89.7	39	100	26	66.7	–	–	–	–
**FARMS VACCINATING**												
PRRSv negative (*n* = 23)	–	–	4	17.4	12	52.2	–	–	–	–	4	17.4
PRRSv positive vaccinated (*n* = 19)	19	100	12	63.2	19	100	–	–	–	–	12	63.2
PRRSv positive unvaccinated (*n* = 9)	–	–	4	44.4	5	55.6	–	–	–	–	2	22.2
SIV negative (*n* = 11)	0	0	–	–	5	45.5	–	–	0	0	–	–
SIV positive vaccinated (*n* = 20)	14	70.0	20	100	18	90.0	–	–	14	70.0	–	–
SIV positive unvaccinated (*n* = 23)	10	43.5	–	–	19	82.6	–	–	10	43.5	–	–
MHYO negative (*n* = 10)	0	0	3	30.0	–	–	0	0	–	–	–	–
MHYO positive vaccinated (*n* = 39)	22	56.4	19	48.7	39	100	14	35.9	–	–	–	–

a Estimated within each row.

b PRRSv negative, PRRSv positive unvaccinated, SIV negative, SIV positive unvaccinated and MHYO negative farms did not vaccinated against PRRSv, SIV or MHYO, respectively.

c PRRSv positive and SIV positive vaccinated gestating sows for PRRSv or SIV, respectively at ~60–80 days of gestation with a single dose. Maiden gilts were vaccinated before entering the breeding herd.

d *MHYO positive farms vaccinated pigs at weaning (i.e., 28 days of age) with a single dose*.

### Production Parameters

Performance information was retrieved from the Teagasc e-Profit Monitor for each of the 56 participating farms. The Teagasc e-Profit Monitor is an online financial analysis tool for assessing farm profitability which contains biological and economic records. In 2017, it included data from 107 herds representing 79,000 sows or 53% of the Irish commercial sow herd. For each group of farms in the different scenarios, performance indicators such as farrowing rate, litters per sow per year, average number of piglets born alive per litter, culling rate, mortality rates for different production stages, age at sale, live weight at sale and kill out (i.e., carcass yield) percentage were obtained for the year 2017 and used to parameterise the bio-economic model.

### Bio-Economic Model

The Teagasc Pig Production Model [TPPM; Calderón Díaz et al. (26)] was used to simulate the effect of herd status and vaccination practice for PRRSv, SIV and MHYO on farm net profit. The TPPM is a stochastic budgetary simulation bio-economic model developed in Microsoft Excel, for farrow-to-finish pig farms with weekly farrowing batches. The TPPM integrates biological, physical and technical parameters and financial analysis and allows the user to investigate the impact of changes in pig production systems on farm performance and profitability. Inputs to the model include biological parameters such as herd size, conception and farrowing rate, number of litters per sow per year, number of piglets born alive per litter and mortality rate for each production stage. Information on reproduction (e.g., number of services and number of boars for heat detection), labor (e.g., number of employees and number of hours worked per week), infrastructure (e.g., number of spaces per stage, energy usage, manure handling) and income (e.g., finisher and culled sow sales) and their associated costs are included as inputs for the TPPM. These inputs are used to calculate physical (e.g., feed usage and number of pigs slaughtered) and financial (e.g., annual cash flow and profit and loss account) outputs. Variable and fixed costs and sales are simulated based on current costs and prices. Net profit is calculated on a total farm basis, as well as per pig produced and per kg of carcass sold.

To simulate animal growth during the production stages, the TPPM includes the Gompertz growth function (27) using the formula $BW=\;W_{0}\exp\left[\mu_{0}\left(1-e^{-Dt}\right)/D\right]$; where **BW** = body weight; **W**_**0**_= the value of the growth function at age 0; **μ**_**0**_ = logarithm of the relative growth rate at age 0 and **D** = slope of the logarithm of the relative growth rate. Nutritional requirements (i.e., energy, amino acids, and minerals) vary for each production stage and are estimated following the recommendations from the National Research Council (**NRC**) Nutrient Requirements of Swine (28) [for more information please refer to Calderón Díaz et al. (26)]. Buildings depreciation, long term bank loans, labor requirements, animal health and other costs including electricity, annual subscription to the Environmental Protection Agency, manure handling cost and transport costs to the abattoir are also included in the TPPM. The only source of income in the TPPM is livestock sales including culled sows and slaughtered finisher pigs [Calderón Díaz et al. (26)].

To account for uncertainty, stochastic features are included into the TPPM by performing stochastic simulation by a process of Monte Carlo sampling to determine the influence of variation in biological inputs, feedstuff costs and carcass prices (i.e. stochastic input variables) on farm profitability (i.e., stochastic output variable) using the Microsoft Excel add-in @Risk (29). Minimum, mean and maximum estimates for feedstuff and price per kg of meat produced were generated based on data recorded on the Teagasc pig e-Profit Monitor between the years 2013 to 2017. Minimum, mean and maximum estimates for biological parameters were generated for each variable for each scenario based on the value range obtained from the Teagasc e-profit Monitor for the year 2017 (Supplementary Table 1). To account for possible co-variation between stochastic variables, spearman correlations were estimated in PROC CORR of SAS v9.4 (SAS Inst. Inc., Cary, NC) and they were included during the Monte Carlo simulation; however, correlations were low and were not significantly different from zero. Stochastic variables were tested for normality using the Shapiro test and by examining the normal plot. All variables were normally distributed except for feedstuff prices. For variables normally distributed a Program Evaluation and Review Technique (PERT) distribution was fitted for each of the stochastic variables. A PERT distribution uses the minimum, most likely and maximum values like the triangular distribution; however, values around the mean are more likely to occur as extremes are not emphasized (29). For variables not normally distributed, the distribution fitting function from @Risk was used to determine their appropriate distribution. An exponential distribution was fitted to feedstuff prices. During the Monte Carlo simulation, 10,000 iterations were run for each stochastic variable. The mean results and variation of results around the mean were reported.

**Figure 1:**
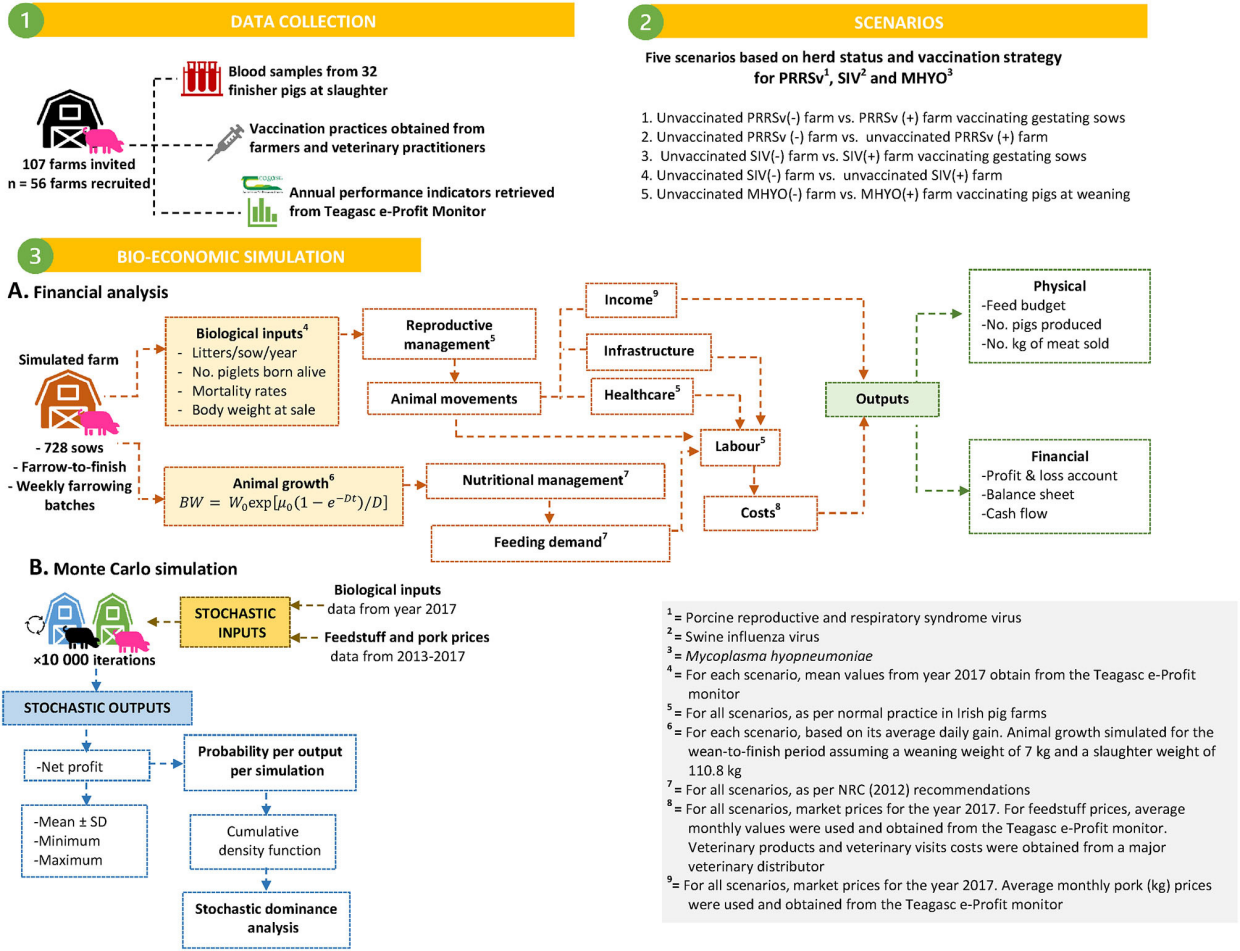
Flow diagram describing data sources and bio-economic modeling process followed to simulate effects associated with different prevalence of pleurisy and lung scars on slaughter pigs on farm performance and profitability. Three scenarios were simulated for a 728 sow farrow-to-finish farm.

In addition, stochastic dominance analysis was carried out performing pairwise comparisons of income distributions for different scenarios being considered by inspecting their cumulative density function (CDF) curve. Stochastic dominance is a partial order of random variables. Scenarios with a CDF further to the right are preferred and thus, the income distribution that exceeds the other, at any level, is stochastically dominant indicating lower financial risk and allowing identification of the preferred scenario (30). When two alternatives *A* and *B*, each with a probability distribution of outcomes *x* (defined by the cumulative probability of *F*_*A*_(*x*) and *F*_*B*_(*x*) are compared, *A* first-order stochastically dominates *B* if *F*_*A*_(*x*) ≤ *F*_*B*_(*x*), for all *x* and there is a strong inequality in at least one point of the distribution. Graphically, the CDF of *A* must always lie below and to the right of the cumulative probability of *B*. Hence, the dominating option *A* is always at least as good an option as *B*. In the event of the second-order stochastic dominance, the CDF of the dominating scenario *A* is still further to the right for the most part of the CDF and more predictable than the dominated alternative *B*, but not for the entire distribution. Formally, the second-order stochastic dominance is examined by comparing the integrals of CDFs (i.e. area under CDF): *A* second-order stochastically dominates *B* if $\int_{-\infty }^{x}F_{A}\left(x\right)dx\leq \int_{-\infty }^{x}F_{B}\left(x\right)dx$ for all *x* with a strict inequality for some range of the distribution. This implies that in a subset of distribution, the dominating alternative *A* may not lead to a better outcome than the dominated alternative *B*. A flow diagram describing data sources and the bio-economic simulation process followed in this study is presented in Figure 1.

### Simulated Farm

For all scenarios, a 728-sow farrow-to-finish pig farm with weekly farrowing batches was simulated. This herd size corresponds to the mean herd size in Ireland for the year 2017 (31). The model was parameterised to simulate mean biological inputs for each scenario from data obtained from the Teagasc e-Profit Monitor (Supplementary Table 1). Farm performance was simulated for an entire year on a weekly basis. Seven animal categories are included in the TPPM which are based on the classification that is general on pig farms in Ireland: (1) piglets (0–4 weeks of age); (2) weaner stage 1 (5–9 weeks of age); (3) weaner stage 2 (10–13 weeks of age); (4) finishers (14–24 weeks of age); (5) maiden gilts (24–32 weeks of age); (6) gestating sows (≥ 32 weeks of age) and (7) lactating sows (≥ 48 weeks of age). Boars (> 10 months of age) used for heat detection were included in the breeding female group as they receive similar feeding and are housed in similar accommodation [for more information please refer to Calderón Díaz et al. (26)]. Number of gilts, gestating and lactating sows as well as number of piglets, weaners and finisher pigs were calculated each week within the TPPM based on the mortality rate for the different production stages. Numbers of culled, dead and slaughtered pigs were also calculated. Maiden gilts and gestating sows were feed restricted, with a common gestation sow diet for 8 weeks and 16 weeks, respectively. Lactating sows were fed *ad libitum* with a common lactating sow diet during the 4-week lactation period. From weaning to slaughter, pigs were fed *ad libitum*. Stage 1 weaner pigs were fed a pre-starter diet during the first week post-weaning, a starter diet during weeks 2 and 3 post-weaning and a weaner diet for 2 weeks. Stage 2 weaner pigs were fed a second weaner diet for 4 weeks and finisher pigs received a finisher diet [see Calderón Díaz et al. (26)].

Replacement gilts were home-reared as per common practice in Irish pig farms (32); selected at 24 weeks of age and artificially inseminated at their second oestrous. All breeding females were artificially inseminated when standing oestrous was observed and 24 h after the first service. Gestating sows were group-housed during the whole gestation period and moved into farrowing accommodation 1 week before their expected farrowing date and remained there for 4 weeks after farrowing when weaning occurred. Prices for PRRSv, SIV, and MHYO vaccines were obtained from a major veterinary distributor in Ireland. Two veterinarian visits per year were considered for the model as per usual practice. Costs for medications (e.g., antibiotics) were not included because of lack of data. Other costs, including electricity use and price per kwh, annual subscription to the Environmental Protection Agency, feedstuff prices, manure handling and transport costs to the abattoir were obtained from the Teagasc pig e-Profit Monitor [for more information please refer to Calderón Díaz et al. (26)]. The only source of income was livestock sales including culled sows and slaughtered finisher pigs. Average daily gain was calculated based on time age at sale using the Gompertz growth curve (27) included in the TPPM and varied according to disease status for each scenario. All pigs were slaughtered once they reached 110.8 kg of body weight which was the average body weight at sale in Irish farms in the year 2017 (31).

## Results

### Porcine Reproductive and Respiratory Syndrome Virus

Compared with PRRSv(-) farms, vacPRRSv(+) farms produced 728 fewer pigs and 60.3 tons less of meat. Also, vacPRRSv(+) farms used 142.2 tons more of weaner feed as animals remained one extra week in the weaner stage of production compared with pigs originating from PRRSv(–) farms (Table 2). Higher variable costs were observed in vacPRRSv(+) farms due to higher weaner feed usage, greater numbers of dead animals for disposal and higher health care costs than PRRSv(–) farms (Table 3 and Supplementary Table 2). Annual sales were €97,824 lower in vacPRRSv(+) farms compared with PRRSv(–) farms. When compared with PRRSv(–) farms, unvacPRRSv(+) farms produced 156 fewer pigs and 12.7 tons less of meat and used 169.7 tons more of weaner feed and 28.5 tons more of finisher feed (Table 2) increasing variable costs (Table 3 and Supplementary Table 1). Annual sales were €19,801 lower when compared with PRRSv(–) farms.

**Table 2:** Average annual physical outputs per simulated scenario from the Teagasc Pig Production Model, a bio-economic model for farrow-to-finish farms developed by Calderón Díaz et al. (26) for the simulation of production effects associated with herd status for porcine reproductive and respiratory syndrome virus (PRRSv), swine influenza virus (SIV), and *Mycoplasma hyopneumoniae* (MHYO).

	**PRRSv**			**SIV**			**MHYO**	
		**Positive**			**Positive**			
**Output**	**Negative^**a**^**	**Vaccinated^**b**^**	**Unvaccinated^**a**^**	**Negative^**a**^**	**Vaccinated^**b**^**	**Unvaccinated^**a**^**	**Negative^**a**^**	**Positive^**c**^**
**Feed usage, ton**								
Gestation feed	540.5	540.5	540.5	540.5	540.5	540.5	540.5	540.5
Lactation feed	367.8	367.8	367.8	367.8	367.8	367.8	367.8	367.8
Creep feed	60.5	59.3	60.1	60.2	59.8	59.9	61.5	59.3
Link feed	142.7	139.8	141.6	142.0	140.9	141.2	144.9	139.8
Weaner feed	916.1	1,058.2	1,085.8	906.4	1,039.5	1,074.9	933.9	1,062.6
Finisher feed	3,380.9	3,308.9	3,409.4	3,345.0	3,617.6	3,391.2	3,452.9	3,327.2
**Sales**								
No. finisher pigs sold	19,136	18,408	18,980	18,928	18,668	18,876	19,552	18,512
No. of tons of meat sold	1,592.8	1,532.5	1,580.1	1,575.5	1,556.1	1,573.5	1,629.5	1,543.1

a PRRSv negative, PRRSv positive unvaccinated, SIV negative, SIV positive unvaccinated and MHYO negative farms did not vaccinated against PRRSv, SIV or MHYO, respectively.

b PRRSv positive and SIV positive vaccinated gestating sows for PRRSv or SIV, respectively at ~60–80 days of gestation with a single dose. Maiden gilts were vaccinated before entering the breeding herd.

c *MHYO positive farms vaccinated pigs at weaning (i.e., 28 days of age) with a single dose*.

**Table 3:** Mean difference in financial accounts between farms with negative (–) and positive (+) status for porcine reproductive and respiratory syndrome virus (PRRSv), swine influenza virus (SIV), and *Mycoplasma hyopneumoniae* (MHYO).

	€**/year**					€**/pig produced**					€**/kg of meat**				
	**PRRSv(+)**		**SIV(+)**		**MHYO^**e**^ (+)**	**PRRSv(+)**		**SIV(+)**		**MHYO (+)**	**PRRSv(+)**		**SIV(+)**		**MHYO (+)**
**Item**	**vac^**b**^**	**unvac^**c**^**	**vac^**d**^**	**unvac^**c**^**		**vac**	**unvac**	**vac**	**unvac**		**vac**	**unvac**	**vac**	**unvac**	
Total Sales	−97824	−19801	−31208	−1720	−140486	+0.1	+0.1	+0.2	+0.3	+0.1	0.00	0.00	0.00	0.00	0.00
**VARIABLE COSTS**															
Feed costs	+17522	+48877	+97842	+53072	−644	+3.9	+3.2	+6.3	+3.0	+4.1	+0.05	+0.04	+0.07	+0.03	+0.05
Other variable costs	+4479	−673	+10975	−51	+16263	+0.6	0.0	+0.7	+0.0	1.4	+0.01	0.00	+0.01	0.00	+0.02
Total variable costs	+22001	+48203	+108817	+53021	+15619	+4.5	+3.2	+7.0	+3.0	+5.5	+0.05	+0.04	+0.08	+0.03	+0.07
Total fixed costs	0	0	0	0	0	+0.5	+0.3	+0.5	+0.3	1.4	0.00	0.00	0.00	0.00	+0.02
Depreciation costs	0	0	0	0	0	+0.5	+0.3	+0.5	+0.3	+0.4	0.00	0.00	0.00	0.00	0.00
					0										0.00
Total costs	+22001	+48203	+108817	+53021	+15619	+5.8	+3.8	+7.5	+3.1	+7.4	+0.07	+0.04	+0.09	+0.04	0.09
Net Profit	−119825	−68004	−140025	−54741	−156106	−5.7	−3.7	−7.2	±2.8	−7.2	−0.07	−0.04	−0.09	−0.03	−0.09

Results^a^ were obtained using Teagasc Pig Production Model, a bio-economic model for farrow-to-finish farms developed by Calderón Díaz et al. (26). In the analysis, farms negative to PRRSv, SIV, and MHYO were assumed as baseline farms, with all results presented with reference to this status.

a An itemized description of sales and variable and fixed costs for PRRSv, SIV and MHYO is available in Supplementary Tables 2–4, respectively.

b Farm vaccinating gestating sows against PRRSv at ~60–80 days of gestation with a single dose. Maiden gilts were vaccinated before entering the breeding herd.

c Positive farms not vaccinating.

d Farm vaccinating gestating sows against SIV at ~60–80 days of gestation with a single dose. Maiden gilts were vaccinated before entering the breeding herd.

e *Farm vaccinating pigs against MHYO at weaning (i.e., 28 days of age) with a single dose*.

**Figure 2:**
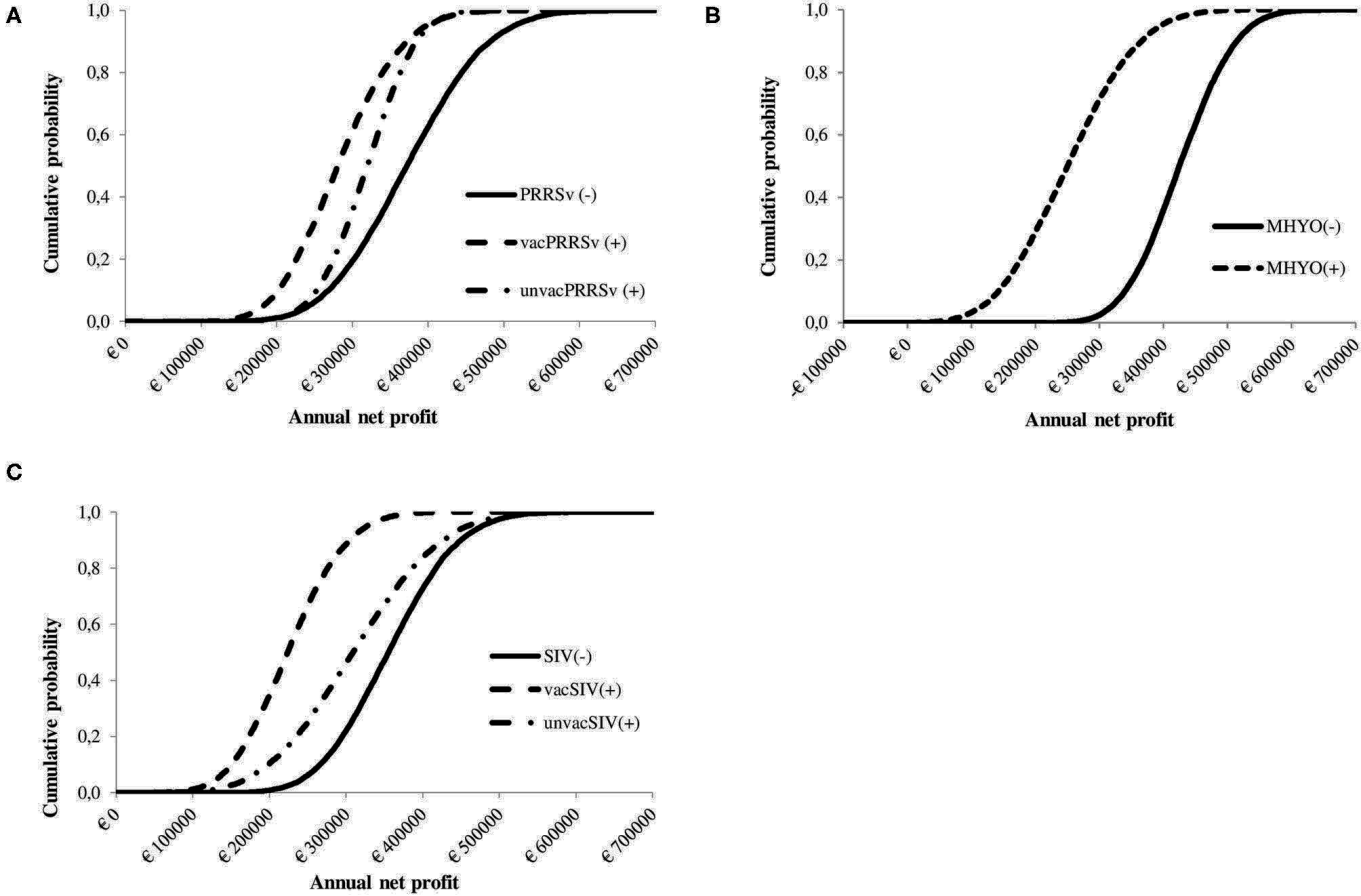
Cumulative probability distribution showing the influence of herd status [i.e., negative (–) or positive (+)] and vaccination practice [i.e., vaccinated or unvaccinated] to **(A)** Porcine reproductive and respiratory syndrome virus [PRRSv(–) not vaccinating, PRRSv(+) vaccinating sows (vacPRRSv) and PRRSv(+) not vaccinating (unvacPRRSv)]; **(B)** Swine influenza virus [SIV(–) not vaccinating vs. SIV(+) vaccinating sows (vacSIV) and SIV(+) not vaccinating (unvacSIV)] and **(C)** *Mycoplasma hyopneumoniae* [MHYO(–) not vaccinating vs. MHYO(+) vaccinating weaner pigs] on farm net profit.

**Figure 3:**
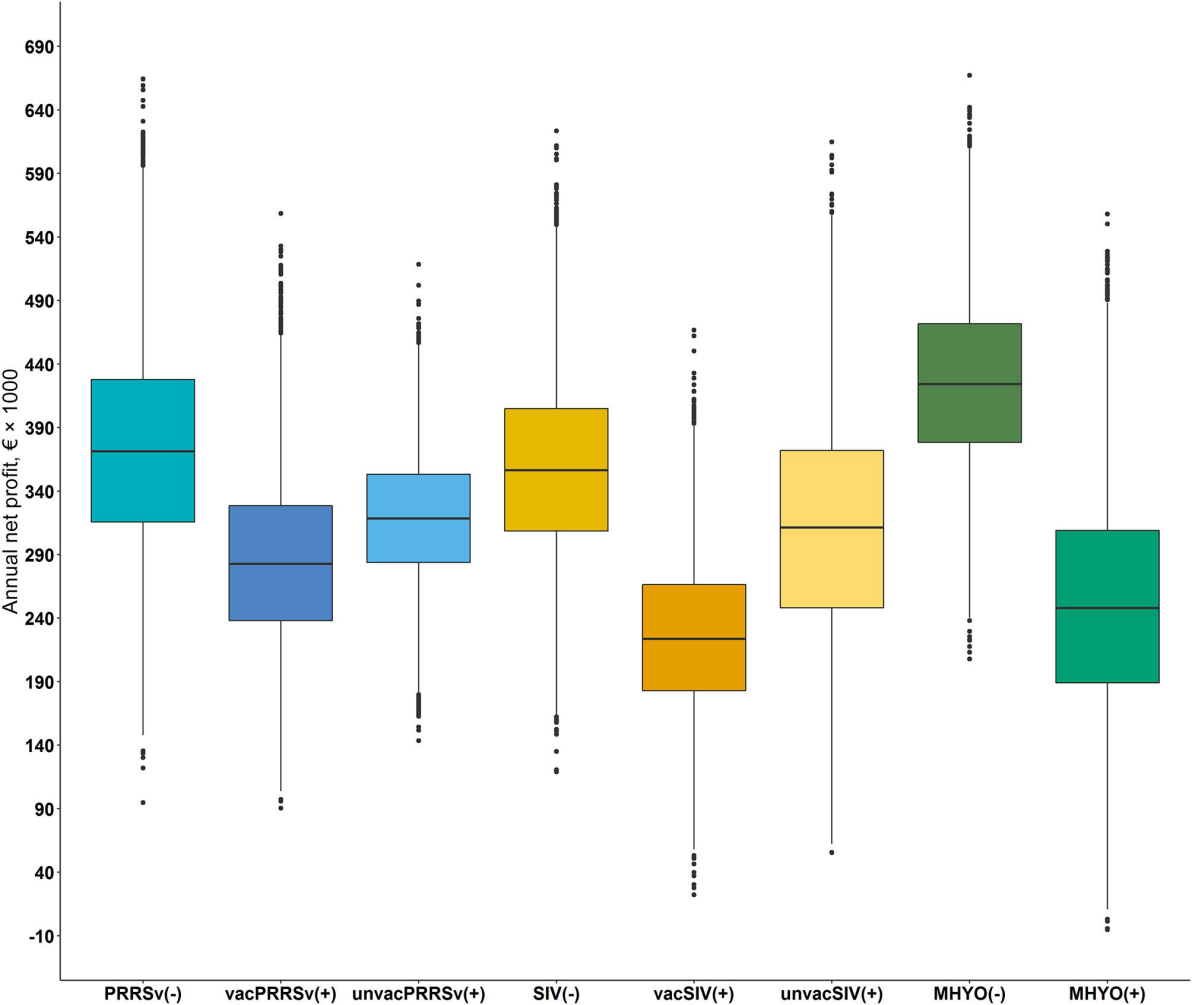
Box and whisker plots of the annual mean net profit, 5th and 95th percentile and the interquartile ranges of farms differing in herd status [i.e., negative (–) or positive (+)] and vaccination practice [i.e., vaccinated or unvaccinated] to Porcine reproductive and respiratory syndrome virus [PRRSv() not vaccinating, PRRSv(+) vaccinating sows (vacPRRSv) and PRRSv(+) not vaccinating (unvacPRRSv)]; Swine influenza virus [SIV() not vaccinating vs. SIV(+) vaccinating sows (vacSIV) and SIV(+) not vaccinating (unvacSIV)] and Mycoplasma hyopneumoniae [MHYO() not vaccinating vs. MHYO(+) vaccinating weaner pigs] on farm net profit.

A cumulative distribution function of the influence of positive status for PRRSv on the spread of farm profit is shown in Figure 2A. The cumulative distribution function shows that PRRSv(–) farms were first order stochastically dominant over vacPRRSv(+) and unvacPRRSv(+) farms. Furthermore, unvacPRRSv(+) farms were second order stochastically dominant over vacPRRSv(+). Mean annual net profit was €373,778 ± 82,309 [90% confidence interval (CI; 5–95%) €242,020 to €513,302] for PRRSv(–) farms; profit per pig produced ranged from €7.74 to €29.26 (mean = €19.38 ± 3.16; 90% CI = €14.00–€24.41) and profit per kg of meat sold ranged from €0.09 to €0.35 (mean = €0.23 ± 0.04; 90% CI = €0.17–€0.29). In vacPRRSv(+) farms, mean annual profit was 24.0% lower (90% CI = €182,017–€396,901) than in PRRSv(-) farms (Figure 3); profit per pig produced ranged from €4.24 to €24.04 (mean = €14.99 ± 2.72; 90% CI = €10.54–€19.46) and profit per kg of meat sold ranged from €0.05 to €0.29 (mean = €0.18 ± 0.03; 90% CI = €0.13–€0.23). In unvacPRRSv(+) farms, mean annual profit was 14.6% lower (90% CI = €234,170–€402,038) than in PRRSv(–) farms; profit per pig produced ranged from €7.37 to €22.42 (mean = €16.41 ± 1.77; 90% CI = €13.37–19.19) and profit per kg of meat sold ranged from €0.08 to €0.28 (mean = €0.20 ± 0.03; 90% CI = €0.16–€0.24).

### Swine Influenza Virus

Weaner and finisher mortality rates were higher in vacSIV(+) farms resulting in 260 fewer pigs sold and 19.4 tons less of meat produced compared with SIV(–) farms (Table 2). Weaner and finisher feed usage was 133.1 tons and 272.6 tons higher in vacSIV(+) farms, respectively, as animals remained for one extra week in each production stage compared with pigs originating from SIV(–) farms (Table 2). Variable costs were higher in vacSIV(+) farms due to higher weaner (+€35,178) and finisher feed (+€63,953) costs, greater numbers of dead animals for disposal (+€1,123) and higher health care costs (+€10,273) compared with SIV(–) farms (Table 3 and Supplementary Table 3). Annual sales were €31,208 lower in vacSIV(+) farms compared with SIV(–) farms (Table 3 and Supplementary Table 3). Similarly, unvacSIV(+) farms sold 52 fewer pigs and 2 tons less of meat than in SIV(–) farms (Table 2) and used 168.4 tons more of weaner feed and 46.2 tons more finisher feed (Table 2) increasing variable costs (Table 3 and Supplementary Table 3). Annual sales were €1,720 lower in unvacSIV(+) farms compred with SIV(–) farms.

The cumulative distribution function shows that SIV(–) farms were first order stochastically dominant over vacSIV(+) and unvacSIV(+) farms (Figure 2B). Additionally, unvacSIV(+) were second order stochastically dominant over vacSIV(+) indicating a lower financial risk for unvacSIV(+) farms. Mean annual net profit was €356,869 ± 71,006 for SIV(–) farms (90% CI of €243,581–€476,810); and it was 36.7 and 12.8% lower in vacSIV(+) and unvacSIV(+) farms, respectively (Figure 3) compared with SIV(–) farms. The 90% CI for mean annual net profit was €131,880–€326,637 for vacSIV(+) farms and €70,833–€457,800 for unvacSIV(+) farms. In SIV(–) farms, profit per pig produced ranged from €9.11 to €27.84 (mean = €18.78 ± 2.79; 90% CI = €14.08–€23.27) and profit per kg of meat sold ranged from €0.11 to €0.33 (mean = €0.23 ± 0.03; 90% CI = €0.17–€0.28). In vacSIV(+) farms, profit per pig produced ranged from €1.63 to €20. 71 (mean = €11.92 ± 2.62; 90% CI = €7.59–€16.17) and profit per kg of meat sold ranged from €0.02 to €0.25 (mean = €0.14 ± 0.03; 90% CI = €0.09–€0.19). Finally, in unvacSIV(+) farms, profit per pig produced ranged from €2.82 to €26.63 (mean = €16.15 ± 3.54; 90% CI = €10.06–€21.71) and profit per kg of meat sold ranged from €0.03 to €0.32 (mean = €0.19 ± 0.04; 90% CI = €0.12–€0.26).

### Mycoplasma hyopneumoniae

Due to less piglets born alive per litter and higher weaner and finisher mortality rates, MHYO(+) farms sold 1,040 fewer pigs and produced 86.4 tons less of meat compared with MHYO(–) farms. Weaner feed usage was 128.7 tons higher in MHYO(+) farms than in MHYO(–) farms as pigs remained one extra week in the weaner stage (Table 2). Variable costs were higher in MHYO(+) farms costs due to a higher weaner feed costs (+€34,200), higher dead animal disposal costs (+€2,935 higher costs) and higher health care costs (+€14,951; Table 3 and Supplementary Table 4). Annual sales were €140,486 less in MHYO(+) farms (Table 3 and Supplementary Table 4).

MHYO(–) farms were first order stochastically dominant over MHYO(+) farms (Figure 2C). Mean annual net profit was 41% lower in MHYO(+) farms (Figure 3) with an associated 90% CI of €113,510–€395,402. The corresponding 90% CI for MHYO(–) farms was €317,184–€537,815. Profit per pig produced ranged from €12.83 to €28.98 (mean = €21.48 ± 2.40; 90% CI = €17.40–€25.27) and profit per kg of meat sold ranged from €0.15 to €0.35 (mean = €0.26 ± 0.03; 90% CI = €0.21–€0.30) in MHYO(–) farms. In MHYO(+) farms, profit per pig produced ranged from -€2.30 to €24.90 (mean = €13.39 ± 3.77; 90% CI = €6.92– €19.31) and profit per kg of meat sold ranged from -€0.03 to €0.30 (mean = €0.16 ± 0.05; 90% CI = €0.08–€0.23).

## Discussion

This study aimed to simulate the bio-economic impact of respiratory disease using field data from commercial farms in a previously constructed stochastic model for farrow-to-finish pig farms. Bio-economic models describe the associations between the components of economic and biological processes (33) and thus, they can be used as tools to understand changes in animal production systems by investigating such associations (34). By parameterising the TPPM with production effects associated with herd status for three respiratory pathogens, we were able to simulate production and financial effects of respiratory disease on intensive integrated pig production systems. Also, by applying stochasticity to the model, the TPPM allowed us to account for the unpredictable and uncertain impact of respiratory disease in different aspects of production. Indeed, respiratory pathogens may have a different effect in different farms due to the inherent differences of individual systems. Thus, by simulating 10,000 iterations the TPPM provides a range of possible outcomes for the economic impact of changes in pig production systems which could prove to be more useful for farmers to make informed decisions regarding disease control.

In this study, financial losses were associated with positive herd status for the three pathogens, with farms positive for MHYO having the greatest losses in annual mean profit. This could be partly due to synergistic effects between respiratory pathogens (35) as bacterial-viral co-infections can exacerbate the pathogenicity of respiratory diseases (35–37). In this study MHYO positive farms were more likely to also be positive to PRRSv and/or SIV than MHYO negative farms and thus, the impact of pathogens co-infections cannot be excluded as a possible reason for the higher financial loses observed in MHYO positive farms. Contrary to the findings reported by Haden et al. (6), we observed higher economic losses associated with MHYO than with PRRSv and this was mainly attributed to lower sales in MHYO(+) farms. Although mean biological parameters used to parameterise the bio-economic model were similar between PRRSv(+) and MHYO(+) farms, the difference for some parameters such as farrowing rate and number of piglets born alive per litter between negative and positive farms was higher for MHYO. This led to a lower number of pigs, and kg of meat sold in MHYO(+) farms, thus increasing financial losses. Another possible explanation for this result could be related to the differences in virulence attributable to the two PRRSv species recognized: the European (Type 1) and the North-American (Type 2) species (38). The North-American species is associated with more severe respiratory disease than the European counterpart (39), which could explain the greater financial impacts of PRRSv compared to MHYO reported in USA studies. Nonetheless, the financial losses reported in this study were likely related to endemic infection. Therefore, results from this study provide information regarding the adverse effects of long-term exposure to these pathogens within Irish pig production systems.

A limitation of our approach is the fact that we could not simulate the financial impact of co-infection among diseases on farm profitability. This was not possible due to the low number of farms negative to all pathogens and the lack of farms positive to only PRRSv or SIV. Studies regarding financial implications of co-infections between respiratory diseases are scarce and mainly done within a single farm [e.g. Haden et al. (6)]. The present study is in line with previous studies investigating the financial implications of respiratory diseases that focus in a single disease at the time [see (8, 40)], due to the complexity of integrating co-infection into the model and the introduction of more uncertainty around the contribution of each pathogen on key biological parameters affected (40). Also, as stated by Nathues et al. (40), it is likely that reduction in the prevalence of a specific pathogen would result in the reduction of secondary infection as pathogens interact and thus, it is questionable if the effects of a single pathogen and co-infection can truly be isolated from each other (40). We acknowledge that the financial impact for each respiratory pathogen investigated in this study was likely affected by other diseases and many other management and environmental issues present on the farms. Nevertheless, the value of this approach is that, contrary to other studies using data from experts and/or data from the literature, this study used actual data from commercial farms and the results provide an indication of the likely financial effects of herd status in European intensive pig production systems. Another limitation is the lack of data on carcass condemnations that are associated with diseases such as MHYO, and thus, kg of meat sold could be overestimated in some cases. Future studies assessing the impact of respiratory disease on carcass condemnations and their financial implications on farm profitability are therefore required. Similarly, additional costs such as costs of antibiotic and other healthcare treatments provided in the different production stages, different management strategies, and extra labor requirements to address respiratory problems in pig farms were not available for inclusion in the TPPM for the financial analysis due to a lack of data. Future studies including this additional aspect are warranted once the necessary information becomes available for inclusion into the TPPM.

An interesting result from this study is that for positive PRRSv and SIV, vaccinating farms had reduced biological performance and lower profit than non-vaccinating farms. Our results more likely reflect the impact of endemic diseases, and thus, vaccines applied in the farms are not preventing the disease but only contributing to reduce it clinical effects. It is possible that farmers vaccinate only when they observe higher mortality rates and reduced (re)productive performance which was the case for all positive farms vaccinating for the PRRSv, SIV and MHYO. It is likely that greater performance and financial losses would be observed in these farms if they were not using vaccines. A limitation of this study is that we did not account for disease dynamics and herd status classification was based on sampling finisher pigs at slaughter. We were not able to establish if virus was actively circulating or in which production stage animals were infected. It is possible that in farms vaccinating against PRRSv, initial infection occurs in earlier production stages and associated negative effects in animal (re)productive performance are also observed in these earlier stages than in positive unvaccinated farms prompting farmers to vaccinate. Similarly, it is possible that positive SIV vaccinating farms experience flu episodes more often than in unvaccinated farms. This would have likely increased financial loses in vaccinating farms. However, this information was not available and warrants further investigation. In spite of this limitation, our results emphasize the importance of implementing disease prevention strategies such as the introduction of a vaccination program against respiratory pathogens when the on-farm prevalence of infected pigs is still low (in the case of positive farms not vaccinating). Also, improved biosecurity measures and improved housing conditions (e.g., reduced stocking density, better ventilation) should be implemented in pig farms.

### Porcine Reproductive and Respiratory Syndrome Virus

In Irish farms, positive status for PRRSv is associated with higher weaner mortality and lower average daily feed intake (Rodrigues da Costa et al., submitted); resulting in fewer pigs produced per sow per year, and pigs requiring more time to reach target slaughter weight. This in turn increased dead animal disposal costs, feed usage and its associated cost during the weaner stages as weaner pigs required one extra week to reach target weight [~38 kg of body weight as per usual practice in Irish pig farms (31)] to be transferred to the finisher stage. Additionally, weaner feed is more expensive than finisher feed as it has higher nutrient concentration as the animal requires more amino acids per kg of body weight to deposit muscle at this stage in their life cycle (41). In fact, increased weaner feed costs represented over 80% of the increase in variable costs in positive farms. These results are in agreement with those reported by Kerkaert et al. (14) for a farm selling weaner pigs at 63 days of age with endemic PRRSv infection where decreased growth rates and increased feed usage were also observed. Additionally, finisher sales were 3.9% lower in vacPRRSv(+) and 0.8% lower in unvacPRRSv(+) farms as fewer pigs were produced and sold per week.

Previous studies estimated economic losses associated with PRRSv in the USA. Holtkamp et al. (8) reported a loss in net profit of US$2.08 (~€1.87) per pig at 120 kg of live body weight in US wean-to-finish production systems and US$2.36 (~€2.07) in farrow-to-weaning production systems. This is lower than the €5.7 and €3.7 per pig produced estimated in the current study in vaccinated and unvaccinated farms, respectively. The difference between studies may be, in part, due to the different production systems (farrow-to-finish in Ireland and multiple site production in the USA) and differences in the cost and revenue structure of production between countries. Our results are similar to those reported by Nathues et al. (40) for a moderate infection affecting performance in the weaner and finisher stages; however, the minimum and maximum farm net profit estimate results for the present study fall within the range of mild to severe infections affecting reproduction and growth performance as reported in the Nathues et al. (40) study. Differences in results are attributable to the modeling approaches. We used actual data to parameterise the bio-economic model whereas Nathues et al. (40) used data reported elsewhere in the scientific literature and expert opinions to parameterise their model. Also, we did not separate the effect of PRRSv on the breeding herd from the effects of the disease during the grow-finisher period but rather analyzed the impact of PRRSv on the whole farm; the impact of PRRSv on reproductive performance is implicit and reflected by the decreased number of pigs born alive in PRRSv positive farms. This finding is in line with the results of Chantziaras et al. (42). Interestingly, positive unvaccinated farms had a higher net profit than vaccinated farms. This suggests that farmers might vaccinate against PRRSv only when they perceive a bigger effect on reduced performance. Indeed, mortality rates in the different production stages were lower in unvacPRRSV(+) farms compared with vacPRRSv(+) farms. It is possible that positive farms that did not vaccinate against PRRSv may implement other measures to control the disease such as improved biosecurity. For example, 85.7% of unvacPRRSv(+) farms always isolated sick pigs and consistently handled them after healthy ones (Calderón Díaz et al., in preparation) compared with 61% of vacPRRSv(+) farms practicing the same measures as per results from the Biocheck.UGent^TM^ (https://www.biocheck.ugent.be/index.php?) scoring tool completed as part of the cross-sectional study conducted by Rodrigues da Costa et al. (32). Additionally, unvacPRRSv(+) farms higher score for disease management than vacPRRSv(+) farms (90.0 ± 21.07 vs. 73.3 ± 20.49, respectively; Calderón Díaz et al., in preparation). Another possible explanation for the better performance of unvaccinated farms is the fact that all PRRSv positive farms that vaccinated were also SIV positive and 94.7% were positive to MHYO and they were twice as likely to be MHYO and SIV positive compared with positive unvaccinated farms. Moreover, PRRSv, positive farms that vaccinated were 1.4, 1.7, and 2.8 times more likely to vaccinate against SIV, MHYO and SIV + MHYO, respectively, than PRRSv positive unvaccinated farms. Co-infection of respiratory pathogens causes more severe disease than single infection (11) and further reduces performances and farm profitability. However, as previously explained, simulation of co-infection scenarios was not possible under the conditions of this study.

### Swine Influenza Virus

Positive herd status for SIV was associated with financial losses in this study. Like PRRSv, farms exposed to SIV had increased feed usage during the weaner and finisher stages increasing feed costs as pigs took longer to reach adequate slaughter weight due to their lower average daily gain (Rodrigues da Costa et al., submitted). These findings are in agreement with previous reports (5). Also, as mortality rates were higher in farms exposed to SIV, they produce fewer piglets per week and therefore, income was reduced. Financial losses per pig produced reported in this study for vacSIV(+) farms (€7.2) are higher than while those in unvacSIV(+) farms (€2.8) are similar to results reported by Haden et al. (6) of US$3.23 (~€2.83). Similar to PRRSv, this is likely due to intrinsic differences in production systems between countries. Like for PRRSv, positive unvaccinated farms had higher net profit than farms that vaccinated. It is likely that farms exposed to SIV but with milder clinical effects, may try to mitigate adverse effects by other means such as improving biosecurity or other aspects of management over vaccination when prevalence is low. This is mostly due to the large genetic diversity of SIV with incomplete cross-protection from one strain to another (43). Indeed, higher biosecurity measures are associated with lower SIV prevalence (44) and in farms with all-in-all-out production systems SIV could disappear as different age groups are not mixed (16). In this study, positive unvaccinated farms had a higher score compared to vaccinated farms (84.2 ± 23.64 vs. 78.0 ± 21.42, Calderón Díaz et al., in preparation) for disease management (including isolation of sick animals and consistently manipulating diseased animals after healthy ones) in the Biocheck.UGent^TM^. It is also possible that the greater financial losses associated with SIV positive vaccinated farms are due, at least in part, to respiratory pathogens co-infection. Although most of positive vaccinated and unvaccinated farms were also positive to MHYO or PRRSv, positive vaccinated farms were 1.7 times more likely to be PRRSv and MHYO positive compared with positive unvaccinated farms. Similarly, SIV positive farms that vaccinated were 1.6 times more likely to vaccinate against PRRSv, 1.1 times more likely to vaccinate against MHYO and 1.6 times more likely to vaccinate against PRRSv + MHYO, respectively, than SIV positive unvaccinated farms. Further studies on the financial implications of co-infections of different respiratory diseases are warranted.

### Mycoplasma hyopneumoniae

*Mycoplasma hyopneumoniae* is one of the main agents in the porcine respiratory disease complex and it is associated with increased healthcare costs, increased mortality and decreased growth performance (7, 45). Positive status to MHYO resulted in higher variable costs, mainly due to increased weaner feed usage as pigs spent one extra week to reach the appropriate weight to be transferred to the next production stage and higher healthcare costs as all weaner pigs were vaccinated. Increased weaner feed and healthcare costs represented 34.6 and 14.4% of the increase in variable costs, respectively. Additionally, MHYO(+) farms had lower total income as they produced fewer tons of meat than negative farms. Positive status for MHYO resulted in a reduction in profit of €5 per pig produced. This is 10 times higher than the losses reported by Haden et al. (6) of US$0.63 (~€0.55). Difference between studies may be because Haden et al. (6) used historical diagnostic reports for pigs originating from the same farm.

Providing animals with appropriate ventilation, temperature and stocking density and all-in-all-out production flow are recommended management practices to control MHYO infections (3). However, according to results from the Biocheck.UGent^TM^ scoring tool, 38.2 and 27.6% of the MHYO(+) participating farms in this study did not follow all-in-all-out management in the weaner and finisher stages, respectively, with routine re-mixing of pigs of different ages occurring regularly in the farms (Calderón Díaz et al., in preparation). Also, MHYO(–) farms had a higher score compared to MHYO(+) farms (86.0 ± 21.05 vs. 80.4 ± 21.62, respectively; Calderón Díaz et al., in preparation) for disease management. Thus, implementing suitable mechanisms of control will be of benefit to infected farms.

Some methodological aspects of the current study are worth noting/considering when interpreting the results. For instance, farms were invited to participate in the study based on the criteria of providing performance records to the Teagasc e-Profit Monitor system. Previous work reported differences between farms recordkeeping in the Teagasc e-Profit Monitor and those that do not participate in the Teagasc e-Profit monitor with regards to welfare indicators [e.g., prevalence of tail lesions; (46)] and thus, it is possible that farms agreeing to participate in this study had better health and performance when compared to farms not participating in the Teagasc e-Profit Monitor. However, the 107 herds providing data to the Teagasc e-Profit Monitor during the year 2017 represented 53% of the national sow herd and a high compliance rate (52%) was obtained among the farmers contacted. Moreover, key performance indicators were similar between participating and non-participating farms recordkeeping in the Teagasc e-Profit Monitor (31). Therefore, the 56 participating farms provide a representative sample of all herds in the Teagasc e-Profit Monitor, and constitute a large sample of 29.2% of Irish national sow herd. Future studies including a larger and more representative sample of Irish pig farms would be advantageous; however, this work provides a good first indication of the financial impact of respiratory pathogens in farrow-to-finish pig farms. Also, there was indication that negative farms and positive unvaccinated farms may rely on improved biosecurity and management strategies to contain disease but we were not able to quantify the associated cost of implementing such measures which could increase production costs and thereby reduce farm profit. Future studies including the necessary investment to improve on-farm biosecurity and its impact on farm profitability would be a natural continuation for this study.

## Conclusions

Farrow-to-finish pig farms with positive status to respiratory disease pathogens, namely PRRSv, SIV and MHYO, suffered financial losses compared to negative herds. Positive status increased feed costs in different production stages for all scenarios simulated. Positive farms had lower annual mean profit and for a given level of profit, risk of financial losses increased with positivity to respiratory pathogens. Contrary to previous reports from other countries, positive status for MHYO was associated with greatest loss in annual mean profit compared with PRRSv. Under the conditions of this study, greater differences were observed between MHYO positive and negative farms for key biological performance indicators than for the other two diseases studied. Additionally, the greater differences observed in MHYO positive farms could be partly due to synergistic effects between respiratory pathogens as MHYO positive farms were more likely to also be positive to PRRSv and/or SIV than MHYO negative farms. In the case of PRRSv and SIV, financial losses were 1.8 and 2.6 times higher in positive vaccinated farms than in positive unvaccinated farms, respectively. This suggests that positive unvaccinated farms rely on management strategies to contain the disease and to minimize its impact while positive vaccinated farms rely more on the vaccine to do the same. The stochastic dominance of negative farms highlights the financial benefits and importance of preventing disease due to respiratory pathogens. Results from this study should encourage farmers to undertake improved disease control measures and/or to implement disease eradication programs to minimize the adverse economic effects of infection with respiratory pathogens. Future studies are needed to investigate the impact of co-infection of different respiratory pathogens on farm performance and farm profitability.

## Data Availability Statement

The datasets used for the results generated for this study are available from the corresponding author upon reasonable request.

## Author Contributions

JACD developed the bio-economic model used for analysis, carried-out the bio-economic analysis and initial drafting of the manuscript. RMF performed serological analysis and collated serological data used to determine disease prevalence. LS and JN contributed to the development of the bio-economic model used for analysis and to outlining the research focus of the article. MRdC collected blood samples and vaccination data, retrieved production parameters from the Teagasc e-profit monitor and constructed the database used to identify the different scenarios used in this study. IK and FCL contributed to outlining the research focus of the article. EGM contributed to the development of the bio-economic model used for analysis and to outlining the research focus and the idea of the article. All authors contributed to preparing the article.

## Conflict of Interest

The authors declare that the research was conducted in the absence of any commercial or financial relationships that could be construed as a potential conflict of interest.
