# Supplementary material for: Financial Analysis of Herd Status and Vaccination Practices for Porcine Reproductive and Respiratory Syndrome Virus, Swine Influenza Virus, and Mycoplasma hyopneumoniae in Farrow-to-Finish Pig Farms Using a Bio-Economic Simulation Model
Source: Front Vet Sci. 2020 Nov 9;7:556674. doi: 10.3389/fvets.2020.556674 (PMC7680737; doi:10.3389/fvets.2020.556674)
Supplement: Supplementary file 1 [file Data_Sheet_1.PDF]

Supplementary Table S1. Biological parameters obtained from the Teagasc e-Profit monitor used to parameterised the Teagasc Pig Production Model, a bio-economic model for farrow-to-finish farms developed by Calderón Díaz et al. (1) to simulate production effects associated with herd status and vaccination practice for porcine reproductive and respiratory syndrome (PRRSv), swine influenza virus (SIV) and *Mycoplasma hyopneumoniae* (MHYO)

	**PRRSv**									**SIV**									**MHYO**					
	**Negative**[1]			**Positive vaccinated**[2]			**Positive unvaccinated**[3]			**Negative**[1]			**Positive vaccinated**[2]			**Positive unvaccinated**[3]			**Negative**[1]			**Positive**[4]		
**Parameters**	**Min**	**Mean**	**Max**	**Min**	**Mean**	**Max**	**Min**	**Mean**	**Max**	**Min**	**Mean**	**Max**	**Min**	**Mean**	**Max**	**Min**	**Mean**	**Max**	**Min**	**Mean**	**Max**	**Min**	**Mean**	**Max**
Farrowing rate, %	73.0	88.4	96.1	78.7	88.6	96.0	81.0	89.3	95.7	73.0	87.7	94.7	79.2	87.9	92.4	78.7	88.6	96.1	81.0	88.4	95.7	73.0	87.8	96.0
Litters/sow/year	2.1	2.3	2.5	2.1	2.3	2.5	2.1	2.3	2.4	2.1	2.3	2.5	2.1	2.3	2.4	2.2	2.3	2.5	2.1	2.3	2.5	2.1	2.3	2.5
No. born alive piglets per litter	12.4	13.7	15.2	12.7	13.5	15.3	13.0	13.4	14.4	12.9	13.7	15.2	12.8	13.6	15.0	11.6	13.5	15.3	13.0	13.9	15.2	11.6	13.5	15.3
Culling rate	34.3	49.4	63.9	37.8	49.3	63.4	38.0	50.3	59.7	38.0	48.2	55.0	37.7	48.4	63.9	34.3	49.9	60.2	38.0	49.7	58.7	34.3	49.1	63.9
Sow mortality rate	1.8	4.3	9.2	2.7	5.1	9.7	2.8	4.8	9.3	2.4	4.6	9.2	2.3	4.9	9.7	2.7	5.3	9.3	2.4	4.6	9.3	1.8	5.1	9.7
Piglet mortality,%	5.6	10.6	15.9	6.1	11.1	14.0	6.8	9.5	12.9	6.8	11.0	15.9	6.1	11.0	14.3	5.6	10.3	15.5	6.8	10.6	14.3	5.6	10.6	15.9
Weaner mortality, %	0.5	2.2	8.9	1.8	3.3	5.1	1.1	2.6	7.1	1.0	2.7	8.8	0.5	3.1	7.1	0.9	2.7	6.8	1.1	2.0	4.3	0.5	3.1	8.9
Finisher mortality,%	1.0	1.9	3.3	1.0	2.3	4.1	0.9	1.6	2.6	0.9	1.9	3.3	1.0	2.2	4.1	1.0	1.9	3.9	0.9	1.6	3.3	1.0	2.1	4.1
Age at sale, days	148.0	168.1	186.0	153.0	176.9	208.0	161.0	177.5	197.0	148.0	166.4	181.0	164.0	179.9	208.0	153.0	172.3	188.0	148.0	168.3	186.0	155.0	176.5	208.0
Kill out %	75.1	76.3	77.8	74.8	76.3	77.6	76.0	76.6	77.4	75.1	76.3	77.8	74.8	76.4	77.4	75.3	76.4	78.1	75.1	76.4	77.8	74.8	76.4	78.1

[1]PRRSv negative, SIV negative, and MHYO negative farms did not vaccinated against PRRSv, SIV or MHYO, respectively

[2]PRRSv positive and SIV positive vaccinated gestating sows for PRRSv or SIV, respectively at approximately 60 to 80 days of gestation with a single dose. Maiden gilts were vaccinated before

[3]PRRSv positive and SIV positive not vaccinating against PRRSv or SIV, respectively.

[4]MHYO positive farms vaccinated pigs for MHYO at weaning (i.e. 28 days of age) with a single dose

Supplementary Table S2. Comparison of trade profit and loss accounts between farms negative to porcine reproductive and respiratory syndrome virus [PPRSv(-)], positive farms vaccinating [vacPRRSv(+)] gestating sows at approximately 60 to 80 days of gestation with a single dose and maiden gilts entering the breeding herd and positive farms not vaccinating [unvacPRRSv(+)]. Results were obtained using Teagasc Pig Production Model, a bio-economic model for farrow-to-finish farms developed by Calderón Díaz et al. (1)

	**€/year**			**€/pig produced**			**€/kg meat sold**		
Item	**PRRSv (-)**	**vacPRRSv (+)**	**unvacPRRSv (+)**	**PRRSv (-)**	**vacPRRSv (+)**	**unvacPRRSv (+)**	**PRRSv (-)**	**vacPRRSv (+)**	**unvacPRRSv (+)**
Sales									
Finisher pigs	2,578,776	2,481,127	2,558,224	134.8	134.8	134.8	1.62	1.62	1.62
Culled sows	43,156	42,981	43,907	2.3	2.3	2.3	0.03	0.03	0.03
Total Sales	2,621,932	2,524,108	2,602,131	137	137.1	137.1	1.65	1.65	1.65
Variable costs									
Gestation feed	126,457	126,432	126,388	6.6	6.9	6.7	0.08	0.08	0.08
Lactation feed	94,748	94,731	94,701	5	5.1	5	0.06	0.06	0.06
Creep feed	53,889	52,789	53,477	2.8	2.9	2.8	0.03	0.03	0.03
Link feed	84,345	82,624	82,624	4.4	4.5	4.4	0.05	0.05	0.05
Weaner feed	238,941	276,617	283,714	12.5	15	14.9	0.15	0.18	0.18
Finisher feed	804,499	787,207	810,852	42	42.8	42.7	0.51	0.51	0.51
Replacement gilts	68,837	68,833	68,827	3.6	3.7	3.6	0.04	0.04	0.04
Dead animal Disposal	9,416	12,483	9,201	0.5	0.7	0.5	0.01	0.01	0.01
Health care	27,019	29,565	26,824	1.4	1.6	1.4	0.02	0.02	0.02
Reproduction	37,309	37,309	37,309	1.9	2	2	0.02	0.02	0.02
Manure handling	16,093	15,639	15,984	0.8	0.8	0.8	0.01	0.01	0.01
Transport	18,183	17,506	18,038	1	1	1	0.01	0.01	0.01
Total variable costs	1,579,735	1,601,736	1,627,938	82.6	87	85.8	0.99	1.05	1.03
Fixed costs									
Admin and accounting	2,500	2,500	2,500	0.1	0.1	0.1	0	0	0
Electricity, heating and light	81,614	81,614	81,614	4.3	4.4	4.3	0.05	0.05	0.05
Insurance	20,533	20,533	20,533	1.1	1.1	1.1	0.01	0.01	0.01
Repairs	20,533	20,533	20,533	1.1	1.1	1.1	0.01	0.01	0.01
Environment	10,000	10,000	10,000	0.5	0.5	0.5	0.01	0.01	0.01
Labour	279,136	279,136	279,136	14.6	15.2	14.7	0.18	0.18	0.18
Loan repayments - of which interest	75,780	75,780	75,780	4	4.1	4	0.05	0.05	0.05
Total fixed costs	490,097	490,097	490,097	25.6	26.6	25.8	0.31	0.32	0.31
Depreciation	175,021	175,021	175,021	9.1	9.5	9.5	0.11	0.11	0.11
Total costs	2,244,852	2,266,853	2,293,056	117.3	123.1	121.1	1.41	1.48	1.45
Net Profit	377,080	257,255	309,076	19.7	14	16	0.24	0.17	0.2

Supplementary Table S3. Comparison of trade profit and loss accounts between farms negative to swine influenza virus [SIV (-)], positive farms to swine influenza virus vaccinating [vacSIV (+)] gestating sows at approximately 60 to 80 days of gestation with a single dose and maiden gilts entering the breeding herd and farms positive swine influenza virus not vaccinating [unvacSIV (+)]. Results were obtained using Teagasc Pig Production Model, a bio-economic model for farrow-to-finish farms developed by Calderón Díaz et al. (1).

	**€/year**			**€/pig produced**			**€/kg meat sold**		
Item	**SIV (-)**	**vacSIV (+)**	**unvacSIV (+)**	**SIV (-)**	**vacSIV (+)**	**unvacSIV (+)**	**SIV (-)**	**vacSIV (+)**	**unvacSIV (+)**
Sales									
Finisher pigs	2,550,746	2,519,364	2,547,541	134.8	135	135	1.62	1.62	1.62
Culled sows	42,108	42,282	43,593	2.2	2.3	2.3	0.03	0.03	0.03
Total Sales	2,592,854	2,561,646	2,591,134	137	137.2	137.3	1.65	1.65	1.65
Variable costs									
Gestation feed	126,473	126,336	126,398	6.7	6.8	6.7	0.08	0.08	0.08
Lactation feed	94,759	94,665	94,708	5	5.1	5	0.06	0.06	0.06
Creep feed	53,614	53,202	53,339	2.8	2.8	2.8	0.03	0.03	0.03
Link feed	83,915	83,269	83,484	4.4	4.5	4.4	0.05	0.05	0.05
Weaner feed	236,455	271,633	279,836	12.5	14.6	14.8	0.15	0.17	0.18
Finisher feed	796,042	859,995	806,565	42.1	46.1	42.7	0.51	0.55	0.51
Replacement gilts	68,839	68,820	68829	3.6	3.7	3.6	0.04	0.04	0.04
Dead animal Disposal	9,995	11118	10,167	0.5	0.6	0.5	0.01	0.01	0.01
Health care	26,889	37,162	26,759	1.4	2	1.4	0.02	0.02	0.02
Reproduction	37,309	37,308	37,309	2	2	2	0.02	0.02	0.02
Manure handling	15,958	15,799	15,925	0.8	0.8	0.8	0.01	0.01	0.01
Transport	17,990	17,748	17,942	1	1	1	0.01	0.01	0.01
Total variable costs	1,568,239	1,677,056	1,621,260	82.9	89.8	85.9	1	1.08	1.03
Fixed costs									
Admin and accounting	2,500	2,500	2,500	0.1	0.1	0.1	0	0	0
Electricity, heating and light	81,614	81,614	81,614	4.3	4.4	4.3	0.05	0.05	0.05
Insurance	20,533	20,533	20,533	1.1	1.1	1.1	0.01	0.01	0.01
Repairs	20,533	20,533	20,533	1.1	1.1	1.1	0.01	0.01	0.01
Environment	10,000	10,000	10,000	0.5	0.5	0.5	0.01	0.01	0.01
Labour	279,136	279,136	279,136	14.7	15	14.8	0.18	0.18	0.18
Loan repayments - of which interest	75,780	75,780	75,780	4	4.1	4	0.05	0.05	0.05
Total fixed costs	490,097	490,097	490,097	25.9	26.3	26	0.31	0.31	0.31
Depreciation	175,021	175,021	175,021	9.2	9.4	9.3	0.11	0.11	0.11
Total costs	2,233,357	2,342,174	2,286,378	118	125.5	121.1	1.42	1.51	1.45
Net Profit	359,497	219,472	304756	19	11.8	16.1	0.23	0.14	0.19

Supplementary Table S4. Comparison of trade profit and loss accounts between farms negative to *Mycoplasma hyopneumoniae* [MHYO (-)] and positive farms [MHYO (+)] vaccinating pigs at weaning (i.e. 28 days of age) with a single dose. Results were obtained using Teagasc Pig Production Model, a bio-economic model for farrow-to-finish farms developed by Calderón Díaz et al. (1)

	**€/year**		**€/pig produced**		**€/kg meat sold**	
Item	**MHYO (-)**	**MHYO (+)**	**MHYO (-)**	**MHYO (+)**	**MHYO (-)**	**MHYO (+)**
Sales						
Finisher pigs	2,638,290	2,498,415	134.9	135	1.62	1.62
Culled sows	43,418	42,806	2.2	2.3	0.03	0.03
Total Sales	2,681,708	2,541,221	137.2	137.3	1.65	1.65
Variable costs						
Gestation feed	126,425	126,424	6.5	6.8	0.08	0.08
Lactation feed	94,726	94725	4.8	5.1	0.06	0.06
Creep feed	54,714	52,789	2.8	2.9	0.03	0.03
Link feed	85,636	82,624	4.4	4.5	0.05	0.05
Weaner feed	243,538	277,738	12.5	15	0.15	0.18
Finisher feed	821,413	791,508	42	42.8	0.5	0.51
Replacement gilts	68,832	68,832	3.5	3.7	0.04	0.04
Dead animal Disposal	8,513	11,448	0.4	0.6	0.01	0.01
Health care	27,409	42,360	1.4	2.3	0.02	0.03
Reproduction	37,309	37,309	1.9	2	0.02	0.02
Manure handling	16,353	15,698	0.8	0.8	0.01	0.01
Transport	18,570	17,603	0.9	1	0.01	0.01
Total variable costs	1,603,439	1,619,059	82	87.5	0.98	1.05
Fixed costs						
Admin and accounting	2,500	2,500	0.1	0.1	0	0
Electricity, heating and light	81,614	81,614	4.2	4.4	0.05	0.05
Insurance	20,533	20,533	1.1	1.1	0.01	0.01
Repairs	20,533	20,533	1.1	1.1	0.01	0.01
Environment	10,000	10,000	0.5	0.5	0.01	0.01
Labour	279,136	279,136	14.3	15.1	0.17	0.18
Loan repayments - of which interest	75,780	75,780	3.9	4.1	0.05	0.05
Total fixed costs	490,097	490,097	25.1	26.5	0.3	0.32
Depreciation	175,021	175,021	9	9.5	0.11	0.11
Total costs	2,268,557	2,284,176	116	123.4	1.39	1.48
Net Profit	413,151	257,045	21.1	13.9	0.25	0.17
